# Successful Post Mortem Caesarian Section

**Published:** 1916-03

**Authors:** 


**Successful Post Mortem Caesarian Section.** J. F. Barnhill reports in the Ind. Med. Jour., Dec. 1915, a case seen in consultation with Dr. W. G. Kelly and operated on by Dr. Hatfield. The mother, aged 36, was 7-months pregnant with her fourth child, dying of cerebro-spinal meningitis secondary to aural infection, pneumococcic and of only two days' duration. Section was performed 5 minutes after death, a male child being delivered alive and surviving at least 24 days, to the date of the report. Quotation is also made from a report by A. E. Mack of Omaha in the J. A. M. A., Aug. 28, 1915. The mother was 36, within a week of full term with her fourth child. She died suddenly of what was determined at necropsy to be pulmonary embolism. The child was apparently dead when delivered shortly after the mother's death, artificial respiration and alternate applications of hot and cold water were unsuccessful, but resuscitation succeeded after injection of epinephrin into the stump of the cord. The child was alive and healthy 7 months after delivery.

